# Supplementary material for: KIBRA controls exosome secretion via inhibiting the proteasomal degradation of Rab27a
Source: Nat Commun. 2019 Apr 9;10:1639. doi: 10.1038/s41467-019-09720-x (PMC6456494; doi:10.1038/s41467-019-09720-x)
Supplement: Supplementary file 1 — Supplementary Information [file 41467_2019_9720_MOESM1_ESM.pdf]

SUPPLEMENTARY FIGURES

Title: KIBRA controls exosome secretion via inhibiting the proteasomal degradation of Rab27a

First author: Song et al.

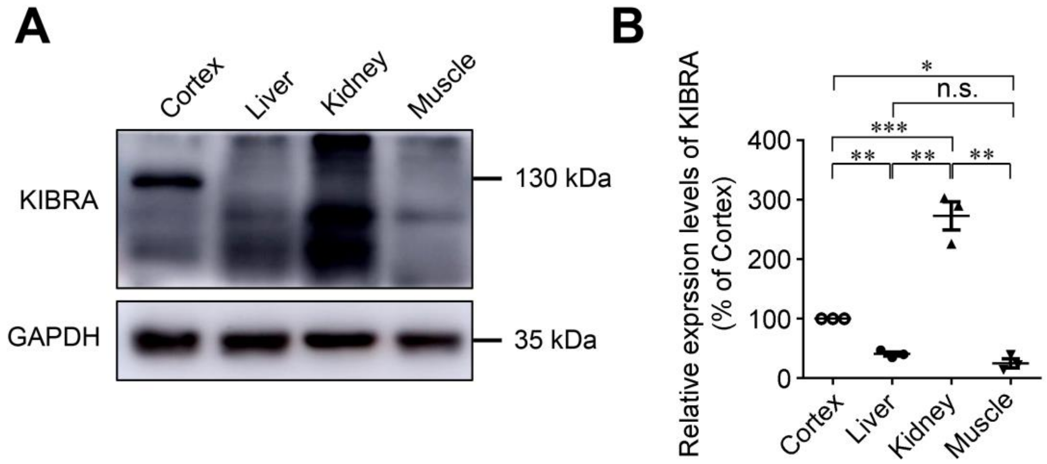

Supplementary Figure 1. Protein levels of KIBRA in different tissues of wild-type mice. **(A)** Western blot analysis of KIBRA in the cortex, liver, kidney, and muscle of wild-type mice (n=3 in each group). (**B**) Quantification of KIBRA protein levels in different tissues of wild-type mice. Quantification results were plotted as dot plots, showing the mean ± SE of three independent experiments. *$P < 0.05$, **$P < 0.01$, ***$P < 0.001$, n.s., not significant ($P > 0.05$) as determined by the one-way ANOVA test.

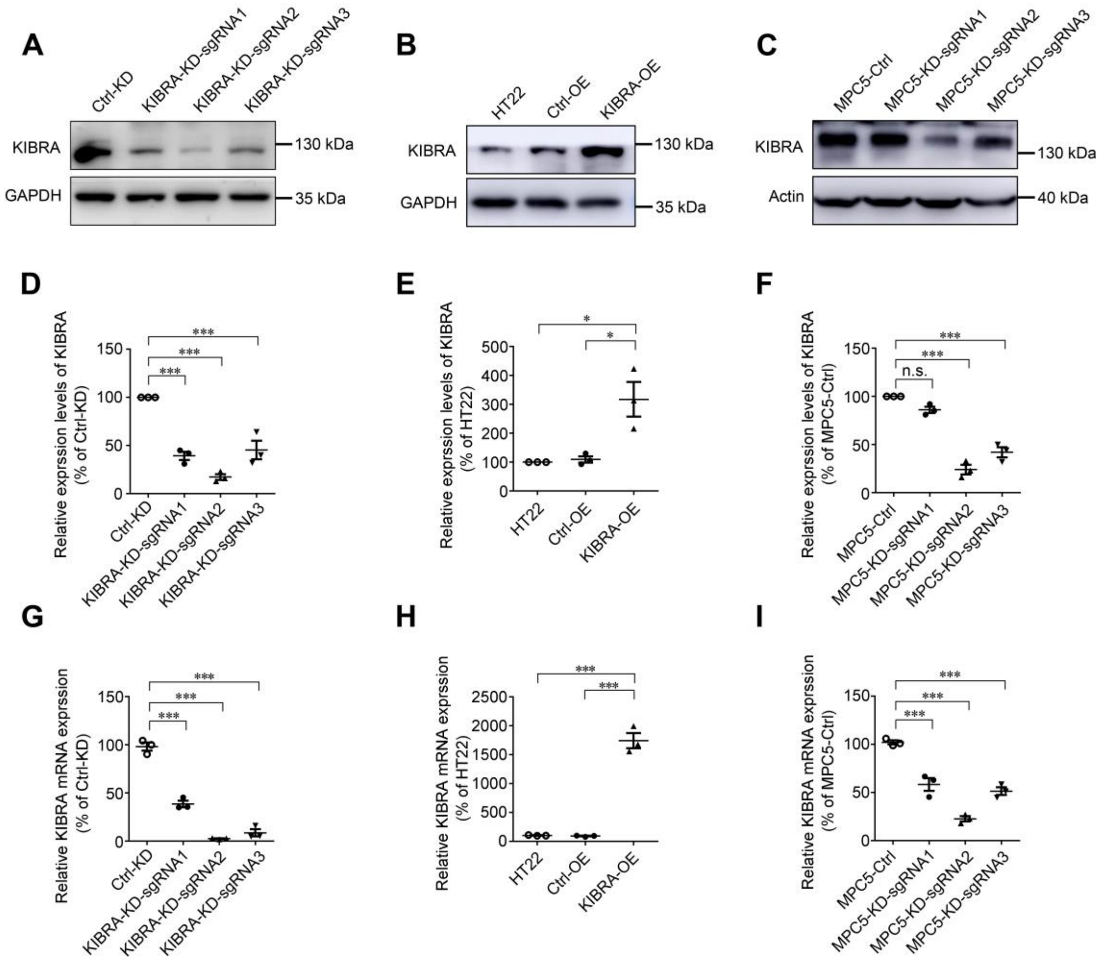

Supplementary Figure 2. Characterization of KIBRA-knockdown and -overexpressed cell lines. (**A**) Western blot analysis of KIBRA in HT22 cell lines stably expressing either control sgRNA (Ctrl-KD) or three different sgRNAs targeting KIBRA (KIBRA-KD-sgRNA1, -sgRNA2, and -sgRNA3). (**B**) Western blot analysis of KIBRA in HT22 cell lines stably expressing empty vector (Ctrl-OE) or wild-type full-length mouse KIBRA for overexpression (KIBRA-OE). (**C**) Western blot analysis of KIBRA in MPC5 cell lines stably expressing either control sgRNA (MPC5-Ctrl) or three different sgRNAs targeting KIBRA (MPC5-KD-sgRNA1, -sgRNA2, and -sgRNA3). (**D-F**) Quantification of KIBRA protein levels in KIBRA-KD (**D**), KIBRA-OE (**E**), KD-MPC5 (**F**) cells compared with their control cells in three independent experiments. (**G-I**) Relative mRNA levels of KIBRA in KIBRA-KD (**G**), KIBRA-OE (**H**), KD-MPC5 (**I**) cells compared with their control cells in three independent experiments. Quantification results were plotted as dot plots, showing the mean ± SE of three independent experiments. $^{*}P < 0.05$, $^{**}P < 0.01$, $^{***}P < 0.001$, n.s., not significant ($P > 0.05$) as determined by the one-way ANOVA test.

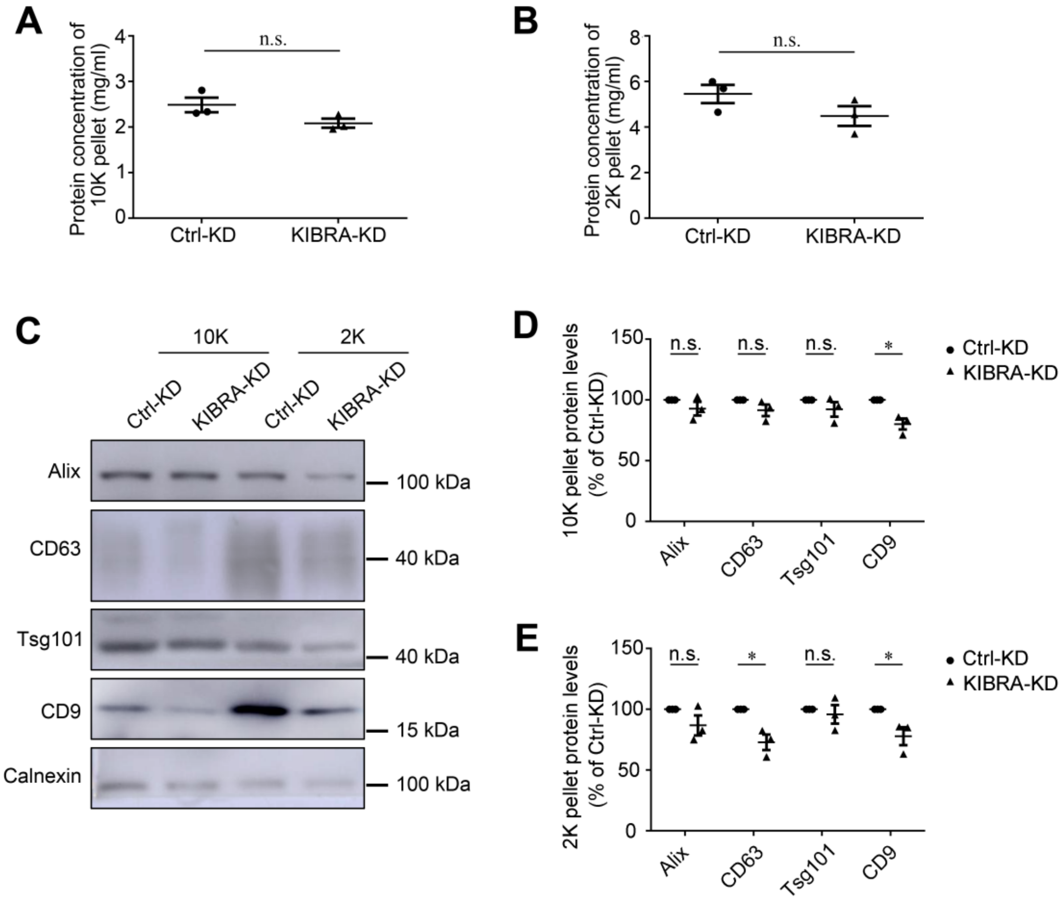

Supplementary Figure 3. Effect of KIBRA on secretion of large EVs in 2K and 10K pellets. (**A**, **B**) Protein concentrations of 10K (**A**) and 2K (**B**) pellets of KIBRA-KD cells and Ctrl-KD cells. 2K and 10K pellets were recovered respectively by low (2,000 g=2K) or medium (10,000 g=10K) centrifugation speed from cell culture supernatants of 20 million cells and resuspended in 60 μl PBS. (**C**) 10K and 2K pellets from equal numbers of KIBRA-KD cells and Ctrl-KD cells were blotted for the exosomal markers Alix, CD63, TSG101, and CD9, and for the endoplasmic reticulum marker Calnexin. (**D**, **E**) Quantification of protein levels of 10K (**D**) and 2K (**E**) pellets obtained from KIBRA-KD cells and Ctrl-KD cells in three independent experiments. The quantification results were plotted as dot plots, showing the mean ± SE of three independent experiments. *$P < 0.05$, n.s., not significant ($P > 0.05$) as determined by two-tailed t-test.

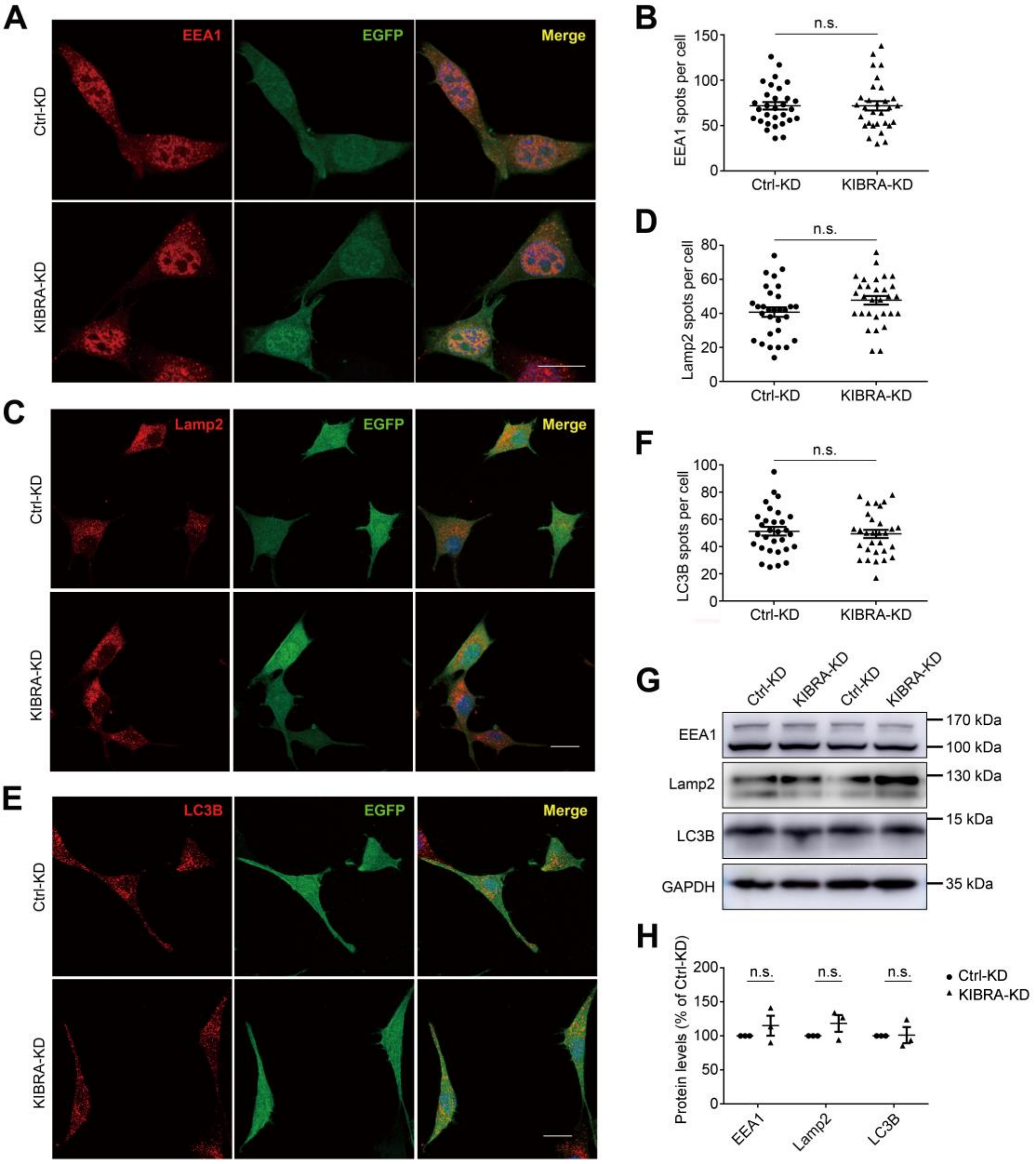

Supplementary Figure 4. KIBRA exerts no significant effect on early endosomes, lysosomes, and autoahagosomes. (**A, C,** and **E**) Confocal microscopy analysis of KIBRA-KD cells and Ctrl-KD cells stained with anti-EEA1 (red, **A**), anti-Lamp2 (red, **C**), anti-LC3B (red, **E**), and DAPI (blue). Cells that were transfected with LV-sgRNA (KIBRA-KD cells) or control vectors (Ctrl-KD cells) could stably express EGFP (green). Scale bar = 10 μm. (**B, D,** and **F**) Quantification of the number of EEA1$^+$ (**B**), Lamp2$^+$ (**D**), and LC3B$^+$ (**F**) particles per cell (n=30). (**G, H**) Western blot analysis (**G**) and quantification (**H**) of EEA1, Lamp2, and LC3B in KIBRA-KD and Ctrl-KD cells in three independent experiments. The quantification results were plotted as dot plots, showing the mean ± SE of three independent experiments. n.s., not significant ($P > 0.05$) as determined by two-tailed t-test.

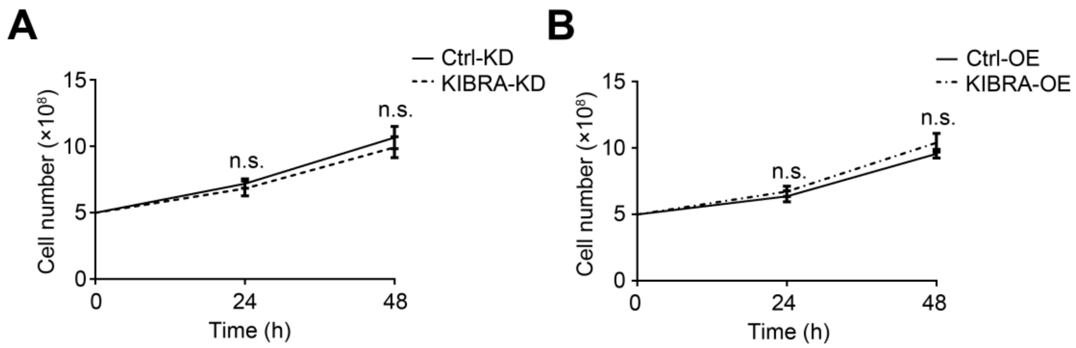

Supplementary Figure 5. Growth rate of KIBRA-KD and KIBRA-OE cells. (**A**, **B**) Numbers of KIBRA-KO (**A**) and KIBRA-OE (**B**) cells compared with their control cells at the time of planting (0 h), and 24 h and 48 h after planting in DMEM medium containing 10% exosome-depleted FBS. The mean ± SE of the three independent experiments were present as line charts. n.s., not significant ($P > 0.05$) as determined by two-tailed t-test.

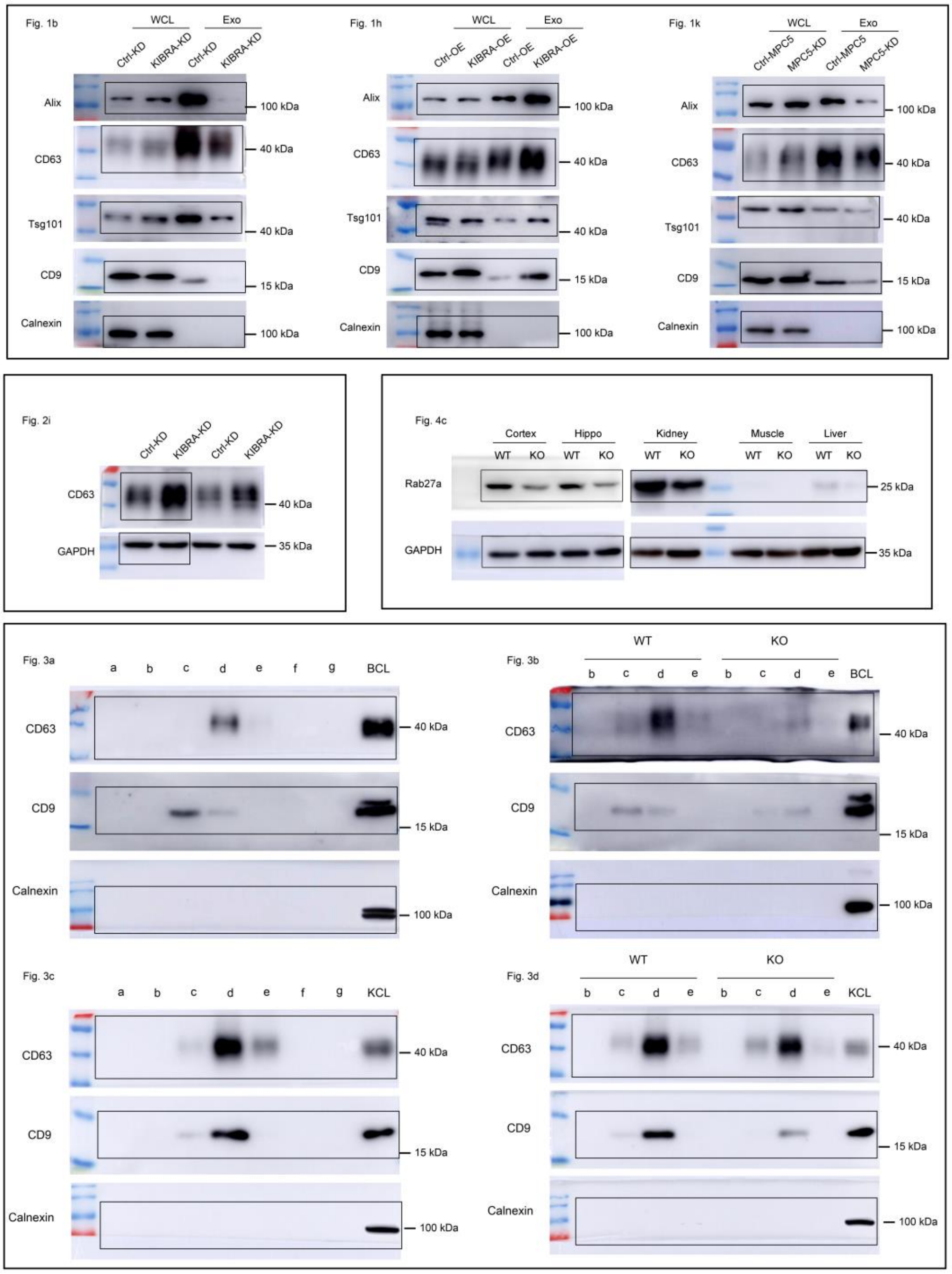

Supplementary Figure 6A. Uncropped Western blots. Full Western blots with indicated areas of selection were presented respectively.

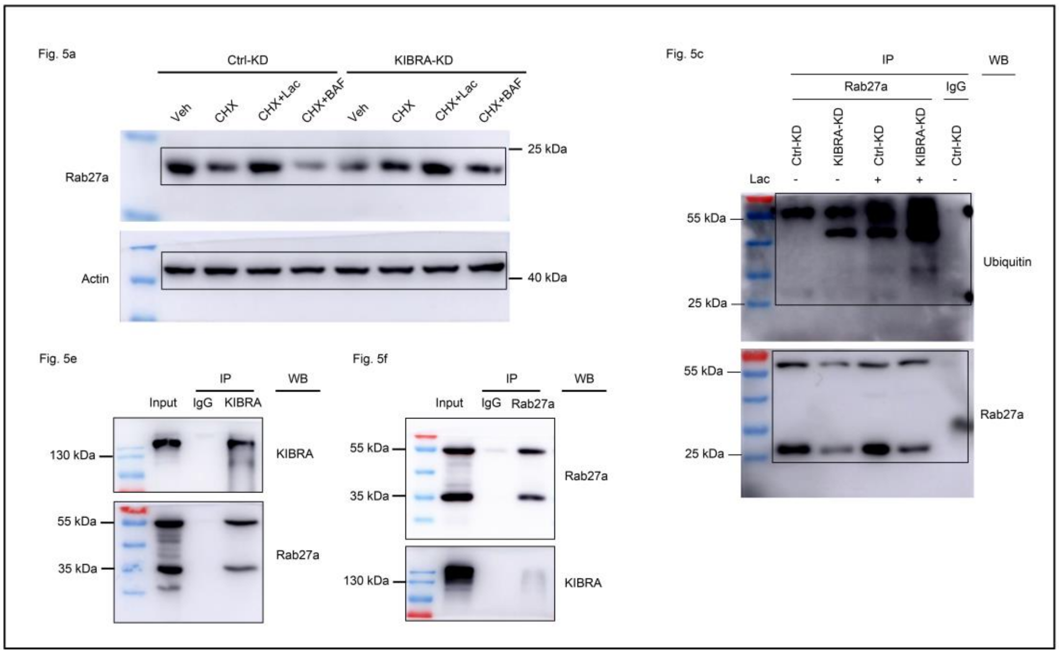

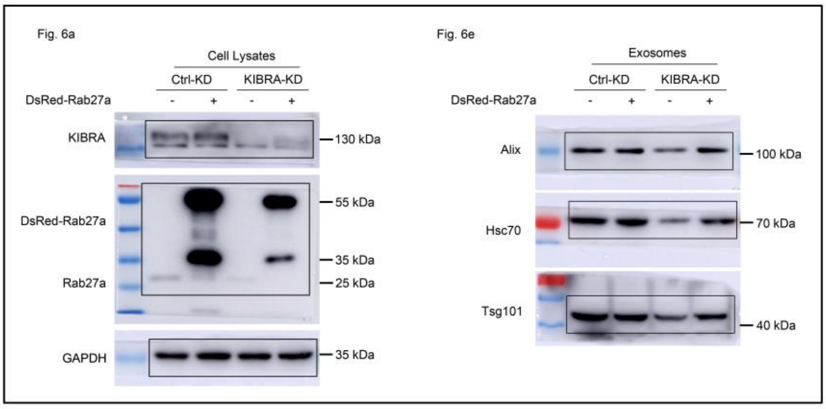

Supplementary Figure 6B. Uncropped Western blots. Full Western blots with indicated areas of selection were presented respectively.

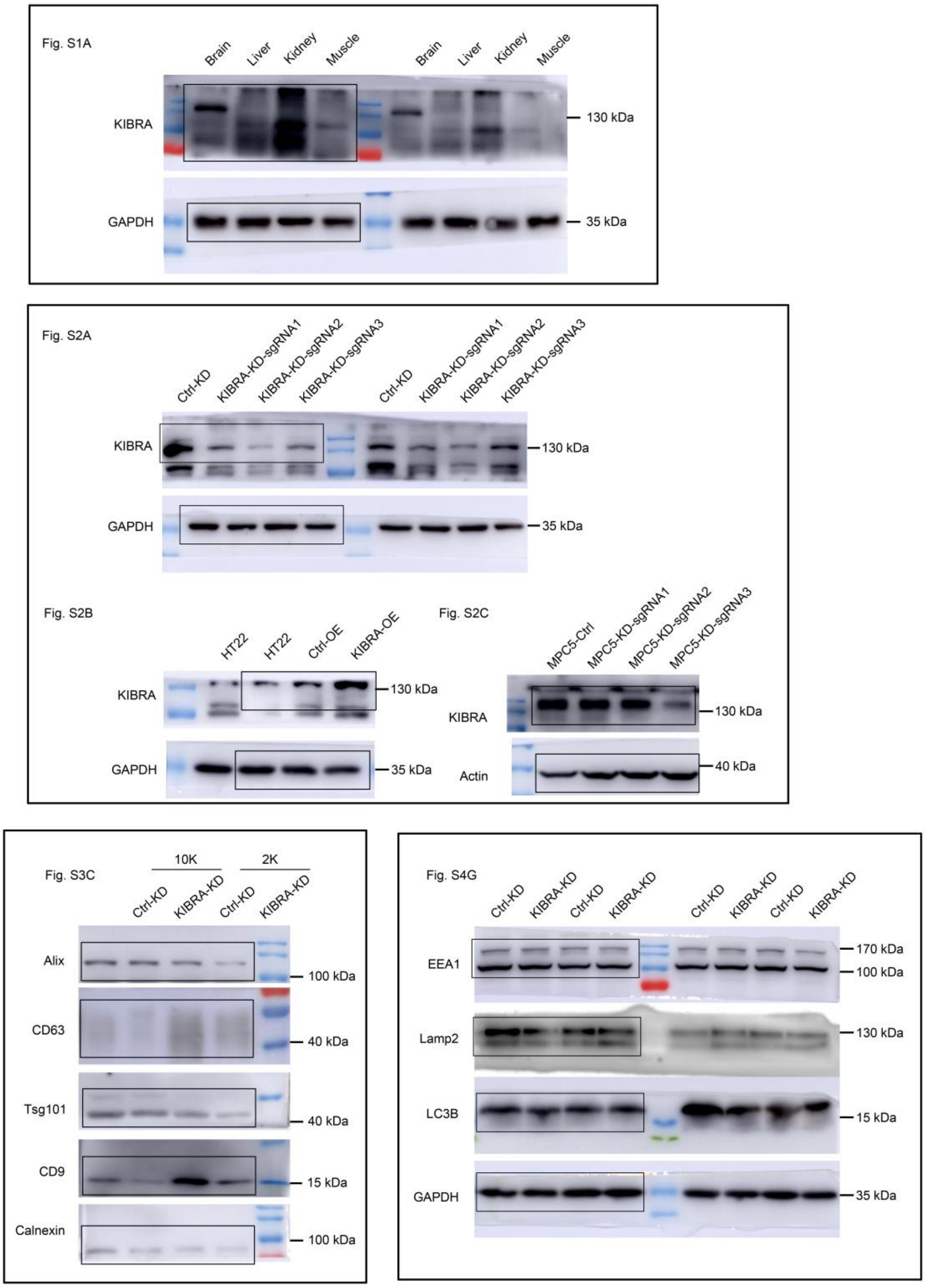

Supplementary Figure 6C. Uncropped Western blots. Full Western blots with indicated areas of selection were presented respectively.

Supplementary Table 1. MS and iTRAQ results of proteins involved in exosome biogenesis and/or secretion

Proteins	WT mice (mean ± SD)	KO mice (mean ± SD)	KO vs. WT log_2FC	KO vs. WT P value
**ESCRT-dependent mechanism**				
HRS	98.70 ± 1.84	102.80 ± 1.61	0.059	0.076
STAM	101.75 ± 4.31	102.17 ± 5.56	0.006	0.935
Tsg101	107.60 ± 2.69	97.37 ± 1.75	-0.144	0.013 *
CHMP4B	97.35 ± 5.87	96.30 ± 2.52	-0.016	0.791
Alix	97.10 ± 1.70	100.50 ± 0.53	0.050	0.198
VPS4A	106.05 ± 4.88	89.87 ± 0.91	-0.239 #	0.127
**ESCRT-independent mechanism**				
Flotillin-2	98.85 ± 2.33	98.9 ± 0.26	0.001	0.981
PLD2	101.70 ± 4.67	98.67 ± 3.18	-0.044	0.440
CD81	104.75 ± 3.75	95.53 ± 4.27	-0.133	0.091
CD9	94.95 ± 4.45	103.47 ± 2.91	0.124	0.076
CD82	98.60 ± 5.37	102.63 ± 0.81	0.058	0.479
**RAB family**				
Rab1a	102.15 ± 3.75	100.83 ± 1.01	-0.019	0.707
Rab2a	103.05 ± 6.29	101.37 ± 2.55	-0.024	0.690
Rab5a	101.25 ± 0.64	101.50 ± 0.36	0.004	0.602
Rab5b	109.65 ± 6.43	95.43 ± 1.76	-0.200	0.183
Rab5c	101.10 ± 11.88	98.53 ± 2.02	-0.037	0.811
Rab7a	102.70 ± 1.98	101.90 ± 1.56	-0.011	0.644
Rab7b	91.90 ± 2.26	107.67 ± 10.03	0.228 #	0.129
Rab9	102.45 ± 1.91	98.00 ± 2.69	-0.064	0.141
Rab11b	97.20 ± 9.48	101.33 ± 2.48	0.060	0.647
Rab14	98.45 ± 1.06	102.10 ± 5.20	0.053	0.420
Rab27b	103.25 ± 0.21	97.07 ± 2.54	-0.089	0.051
Rab27a	106.45 ± 3.32	90.30 ± 2.74	-0.237 #	0.009 **
Rab31	105.20 ± 5.66	100.43 ± 1.65	-0.067	0.437
Rab35	93.40 ± 4.53	102.77 ± 0.67	0.138	0.205
**SNARE family**				
VAMP2	98.85 ± 2.47	104.60 ± 5.94	0.082	0.301
VAMP3	114.80 ± 10.47	96.77 ± 13.54	-0.247 #	0.215
VAMP7	95.70 ± 4.95	100.17 ± 0.25	0.066	0.423
YKT6	98.80 ± 0.85	98.03 ± 0.91	-0.011	0.422
Vti1b	94.40 ± 1.56	103.13 ± 1.80	0.128	0.012 *
Syntaxin1A	100.80 ± 13.15	100.03 ± 1.59	-0.011	0.948
**Other proteins**				
VTA1	101.80 ± 7.92	98.67 ± 1.37	-0.045	0.675
Syntenin	106.35 ± 8.84	97.77 ± 7.15	-0.121	0.312
Syndecan	98.30 ± 4.24	102.60 ± 3.86	0.062	0.323

Synaptotagmin-7	103.00 ± 0.57	102.07 ± 5.35	-0.013	0.831
Rabgap1	105.80 ± 2.69	94.03 ± 1.80	-0.170	0.009**
RalA	99.25 ± 1.91	100.13 ± 2.81	0.013	0.729
RalB	100.80 ± 7.35	103.10 ± 5.67	0.033	0.715
Cortactin	96.80 ± 6.65	104.93 ± 0.95	0.116	0.331
ARF6	107.80 ± 3.68	100.30 ± 1.61	-0.104	0.046*
PKM	101.95 ± 0.07	101.00 ± 1.40	-0.014	0.430

*P < 0.05, **P < 0.01 as determined by two-tailed t-test. # $|\log_2 FC| > 0.2$.
